# Infliximab concentration monitoring improves the control of disease activity in rheumatoid arthritis

**DOI:** 10.1186/ar2867

**Published:** 2009-11-25

**Authors:** Denis Mulleman, Jean-Camille Méric, Gilles Paintaud, Emilie Ducourau, Charlotte Magdelaine-Beuzelin, Jean-Pierre Valat, Philippe Goupille

**Affiliations:** 1 Université François-Rabelais de Tours, 3 rue des Tanneurs, 37041 Tours Cedex 1, France; 2 Centre Hospitalier Régional et Universitaire de Tours, Service de Rhumatologie, avenue de la République, 37044 Tours Cedex 9, France; 3 Centre Hospitalier Régional et Universitaire de Tours, Laboratoire de Pharmacologie-Toxicologie, 2 boulevard Tonnellé, 37044 Tours Cedex 9, France; 4 Centre Hospitalier Régional et Universitaire de Tours, Laboratoire d'Immunologie, 2 boulevard Tonnellé, 37044 Tours Cedex 9, France; 5 Institut national de la santé et de la recherche médicale Centres Investigations Clinique 202, 2 boulevard Tonnellé, 37044 Tours Cedex 9, France

## Abstract

**Introduction:**

Adjustment of infliximab dosage for individuals may be useful in improving therapeutic response in rheumatoid arthritis (RA). Herein, we aimed to determine whether measurement of infliximab serum concentration modifies the therapeutic decision and improves the control of disease activity.

**Methods:**

RA patients routinely treated with infliximab were included in an observational open-label study. On visit 1 (V1), according to the disease activity, a preliminary therapeutic decision was selected among four therapeutic options and a blood sample was collected to measure trough serum infliximab concentration. The final therapeutic decision, based on both disease activity and serum infliximab concentration assessed at V1, was applied at the following infusion (V2). Clinical and biological evaluations were performed at V3 and V4 and compared with those at V1.

**Results:**

We included 24 patients. The final therapeutic decision differed from the preliminary decision for 12 patients (50%). For patients with increased infliximab dosage at V2, mean disease activity score for 28 joints (DAS28) decreased by about 20% at V3 or V4 as compared with V1 (*P *< 0.05). Decreased DAS28 was correlated with increased serum infliximab concentration (*P *< 0.02).

**Conclusions:**

The measurement of infliximab trough concentration modifies the therapeutic decision for RA patients and helps improve control of disease activity. Therapeutic drug monitoring of infliximab in RA may be useful for individual dosage adjustment.

## Introduction

Infliximab is a chimeric monoclonal antibody against tumor necrosis factor-alpha (TNF-α) used in the treatment of rheumatoid arthritis (RA); its efficacy was demonstrated in a randomized controlled trial [1]. The variable inter-individual response is explained at least in part by individual pharmacokinetics [2]. Clinical response in RA is indeed influenced by infliximab serum concentration [2-6], and we have recently shown that this concentration predicts long-term disease control in daily practice [7]. Adjustment of infliximab dosage for individuals may be useful in improving therapeutic response [8,9]. Hence, patients with persistent active disease and low infliximab concentrations could benefit from an increase in infliximab dosage, whereas those with poor disease control and high infliximab concentrations should switch to another biopharmaceutical. In contrast, patients with optimal disease control and high infliximab concentrations might benefit from a controlled reduction in infliximab dose or an increase of dosing intervals to decrease the risk of dose-related side effects.

We aimed to determine whether the measurement of serum infliximab concentration modifies the therapeutic strategy and improves the control of disease activity in RA. The secondary objective was to study whether this improvement in control of disease activity is related to changes in infliximab concentration.

## Materials and methods

### Patients

**Figure 1:**
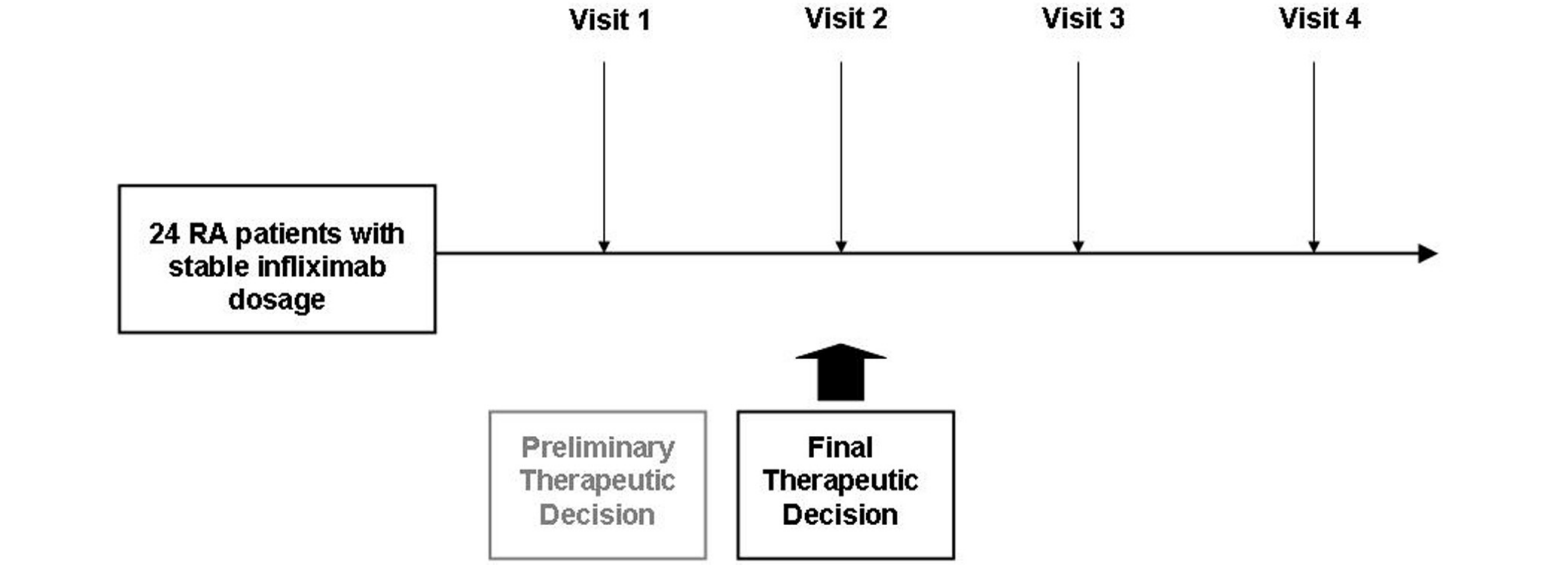
Study design. Each visit corresponds to infliximab infusion and included clinical assessment, a blood sample for measurement of infliximab, and testing for antibody against infliximab. The final therapeutic decision took into account infliximab concentration measured at visit 1. RA, rheumatoid arthritis.

All RA patients visiting our rheumatology department from May to August 2007 to receive their routine infliximab infusion, according to recommendations from the French Society for Rheumatology for patients receiving anti-TNF biopharmaceuticals [10], were followed up during four visits (V1 to V4). According to the rules applied in our setting, patients were asked to complete a weekly questionnaire recording pain, joint stiffness, and disease activity on visual analog scales to estimate overall disease activity since the last infusion. In this observational study, eligible patients had received at least four infusions of infliximab, had an infliximab dose (in milligrams per kilograms) and dosing interval (± 4 days) similar to those of the previous infusion, had no change in use of disease-modifying anti-rheumatic drugs since the previous infusion, and had no pregnancy or surgery scheduled within the following three infusions. The study design is summarized in Figure 1. The patients gave their informed consent to participate in the study, and the study protocol was in compliance with the Declaration of Helsinki. This study was approved by our local institutional review board.

### Serum concentrations of infliximab and antibody toward infliximab

A blood sample was drawn just before each infusion from V1 to V4. Serum infliximab concentrations were measured by enzyme-linked immunosorbent assay (ELISA) as previously described [11]. The limit of detection of this technique is 0.014 μg/mL. The lower limit of quantitation and upper limit of quantitation are 0.04 and 4.5 μg/mL, respectively. Serum concentrations of antibody toward infliximab (ATI) were measured by double-antigen ELISA with capture by infliximab-coated microplates and detection by peroxydase-coupled infliximab. The ELISA was standardized by use of a mouse monoclonal antibody to all subclasses of human IgG. The limit of detection was 0.20 μg/mL. The technique was tested on 195 serum samples from healthy blood donors (37%), patients with autoimmune diseases (59.5%), and patients with hypergammaglobulinemia (3.5%) and did not lead to false-positive results, even in the presence of rheumatoid factor. Because of the interference with circulating infliximab, results were conclusive if the infliximab concentration in the sample was less than 1.7 μg/mL.

### Preliminary therapeutic decision (before knowing infliximab concentration)

The preliminary therapeutic decision at V1 was based on clinical and conventional laboratory marker testing. Control of disease activity was categorized as 'optimal', 'acceptable', or 'inadequate' by a combination of disease activity score for 28 joints (DAS28) and physician global assessment. Taking into account the control of disease activity, the physician selected a preliminary therapeutic decision from among four options: (a) decrease infliximab dosage, (b) maintain the same infliximab dosage and possibly add another therapeutic intervention (that is, intra-articular corticosteroid injection, physical treatment, or analgesic pharmaceuticals), (c) increase infliximab dosage, or (d) discontinue infliximab and switch to another treatment. An increase in infliximab dosage was not possible if the V1 dosage was considered maximal (that is, a dose of at least 7.5 mg/kg or an interval of not more than 4 weeks).

### Final therapeutic decision

The final therapeutic decision applied at V2 (Figure 1) took into account both the assessment of disease activity and the trough serum infliximab concentration measured at V1 (Table 1). According to available published data [2,3,12,13], a target infliximab serum concentration of 5 μg/mL was selected. Trough infliximab concentration was classified as low (<2.0 μg/mL), medium (≥2.0 and <8 μg/mL), or high (≥8 μg/mL).

**Table 1:** Final therapeutic decision at visit 2 based on disease activity control and serum trough infliximab concentration

		Control of disease activity		
		
		Optimal	Acceptable	Inadequate
Infliximab trough concentration	High: C (μg/mL) ≥8.0	Decrease infliximab dosage	Maintain same infliximab dosage	Switch to another biopharmaceutical
	Medium: 2.0 ≤ C (μg/mL) <8.0	Maintain same infliximab dosage	Consider increasing infliximab dosage^a^	
	Low: C (μg/mL) <2.0		Increase infliximab dosage	

Infliximab dosage could not be increased if the current dosage was considered maximal (that is, at least 7.5 mg/kg or interval of not more than 4 weeks). ^a^If the physician kept the same infliximab dosage, another therapeutic intervention could be added (that is, intra-articular corticosteroid injection, physical treatment, or analgesic pharmaceuticals). C, concentration.

### Statistical analysis

Clinical and biological markers of disease activity, DAS28, and trough serum infliximab concentration at V1 were compared with those at V3 and V4. Statistical differences were analyzed by the Wilcoxon non-parametric test for continuous variables. The relation between change in infliximab serum concentration and change in DAS28 from V1 to V4 was analyzed by the Spearman non-parametric correlation test. Statistical analysis involved Statview version 5 (SAS Institute Inc., Cary, NC, USA). A two-tailed *P *value of less than 0.05 was considered statistically significant. Results are presented as median [range] unless otherwise stated.

## Results

### Characteristics of patients

A total of 24 patients (8 men) were included. The median age was 61 years [34 to 78], and the disease duration was 10 years [5 to 58]. The median duration of infliximab treatment was 56 months [3 to 76], and the number of previous infliximab infusions was 31 [4 to 39]. Before receiving infliximab, 2 patients (9%) had received one of the two other anti-TNF-α biopharmaceuticals available at that time. Sixteen patients were receiving prednisone (dose/day = 5 mg [0.5 to 10.0]), and 20 patients were receiving methotrexate (dose = 10 mg/week [5 to 17.5]). The median interval between infliximab infusions was 8 weeks [6 to 9], and the dose of infliximab infusion was 3.75 mg/kg [2.80 to 7.30]. The median infliximab concentration was 3.00 μg/mL [0.01 to 7.80]. No patient had a high trough infliximab concentration, 17 patients (71%) had medium trough concentrations, and 7 (29%) had low trough concentrations. The median erythrocyte sedimentation rate was 14 mm/hour [5 to 49], the level of C-reactive protein was 5.0 mg/L [0.6 to 22.0], and DAS28 was 2.65 [1.48 to 6.16]. At V1, 12 patients (50%) showed optimal control of disease activity, 5 (21%) acceptable control, and 7 (29%) inadequate control. Only one patient was positive for ATI at V1 (2.18 μg/mL) with a concomitant infliximab concentration that was undetectable (0.01 μg/mL). This patient had been receiving infliximab for 9 months. Her infliximab dosage was increased during the study, but she eventually discontinued the treatment after V4.

### Difference between preliminary and final therapeutic decision

The final therapeutic decision, taking into account serum infliximab concentration, differed from the preliminary therapeutic decision for 12 patients (50%) (Additional data file 1). For 7 patients, the infliximab dosage was increased for the final therapeutic decision as compared with 3 patients for whom the dosage was increased at the preliminary therapeutic decision. Of the 7 patients, 6 were considered to have an inadequate control of disease activity (2 with medium and 4 with low infliximab concentration) and 1 was considered to have acceptable control (with medium infliximab concentration).

### Evolution of clinical and biological markers of disease activity

**Figure 2:**
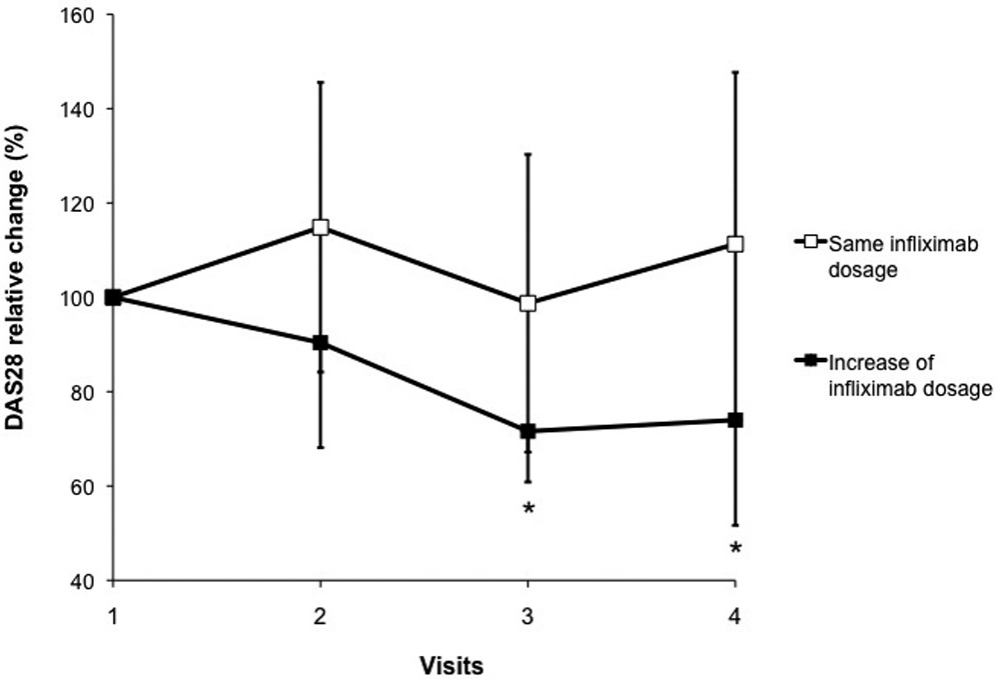
Relative change in mean disease activity score for 28 joints (DAS28) over time according to final therapeutic decision (Table 1). *P < 0.05.

Changes in DAS28 over time are presented in Figure 2. DAS28 significantly improved from V1 to V3 (*P *< 0.05) for patients with an increase in infliximab dosage. The mean improvement in DAS28 for patients of this group was about 20%. The improvement as compared with V1 was maintained at V4 (*P *< 0.05).

### Relation between change in serum infliximab concentration and change in disease activity

**Figure 3:**
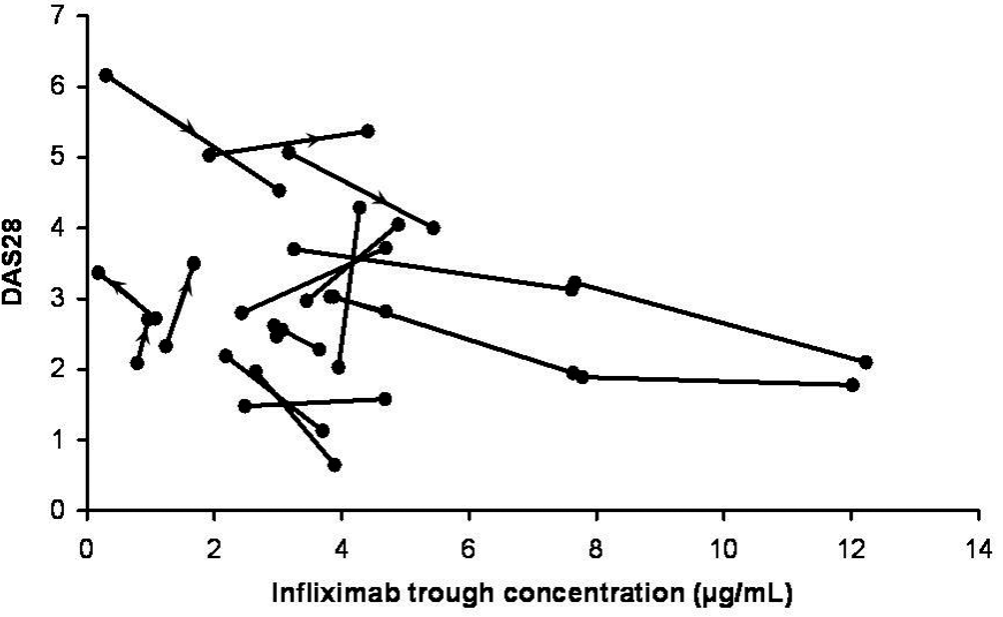
Relation between change in serum infliximab concentration and change in disease activity score for 28 joints (DAS28) from visit 1 to visit 4. Because of missing data, four patients are not represented.

As shown in Figure 3, changes in DAS28 and infliximab concentration between V1 and V4 were inversely related (*P *< 0.02).

## Discussion

To our knowledge, this is the first study to highlight the usefulness of pharmacokinetic monitoring of infliximab concentration to control disease activity in RA. Indeed, the measurement of trough infliximab concentration modified the therapeutic decision for half of our patients and led to improved control of disease activity for patients for whom infliximab dosage was increased. Our results confirm those of a clinical observational study that did not monitor infliximab concentrations [8]. The strength of our approach lies in a strategy that took into account both disease activity and serum infliximab concentration.

The second important finding is the relation between increase in serum infliximab concentration and clinical improvement, as measured by the DAS28. Because concomitant medications remained unchanged during the study for patients whose infliximab dosage had been increased, the improvement in DAS28 should have resulted from the increase in infliximab concentration. Our results also confirm the inter-individual variability of infliximab pharmacokinetics. Despite a median infliximab dose (3.75 mg/kg [2.80 to 7.30]) above that recommended in patients with RA, 7 patients (29%) had low trough concentrations.

Clinicians can face two situations with RA patients receiving infliximab. The first corresponds to patients with optimal disease control, for whom the physician could consider reducing infliximab dosage. However, a decrease in infliximab dosage without infliximab concentration monitoring may lead to underexposure, a situation that can predict insufficient control of disease activity and increased risk of positivity for ATI [4,14]. For the preliminary therapeutic decision, infliximab dosage reduction was planned for 6 patients; however, because these patients were all considered satisfactorily exposed or underexposed, they continued the same regimen for the final therapeutic decision. The second situation corresponds to patients with disease control the physician considers acceptable or inadequate. In this situation, the physician could consider increasing the infliximab dosage to improve the control of disease activity. However, concerns arise in the prolonged use of TNF-α antagonists, especially at high doses [15]. Therefore, the measurement of serum infliximab concentration in this situation could be useful to avoid an unnecessary increase in dosage in patients with concurrent high infliximab concentration and hence a risk of dose-dependent side effects. Monitoring of infliximab concentrations in this situation would lead the clinician to increase the dosage only if the patient does not have a high infliximab concentration. Therapeutic drug monitoring of infliximab could result in a lowering of dosage in patients having higher exposure than needed, in an increasing of dosage in others, or in a discontinuation of infliximab in non-responders with high exposure. As a whole, such a strategy should improve the cost-effectiveness of infliximab in RA.

Our method to determine infliximab serum concentration detects the active form of infliximab (that is, the species that can bind to TNF-α), not infliximab molecules that are already bound to TNF-α or ATI. The choice of measuring the active form allows investigators to analyze the concentration-effect relationship.

Immunogenicity induced by anti-TNF-α biopharmaceuticals has been associated with low concentrations and poor clinical outcomes [16]. Our technique for ATI detection was limited by the presence of circulating infliximab. The problem is well known and is encountered whatever the method used [17]. Also, ATI may have underestimated the infliximab determination by neutralizing both infliximab paratopes. However, such undetectable infliximab molecules are inactive and cannot be taken into account for pharmacokinetic-pharmacodynamic analysis. Positivity for ATI influences the pharmacokinetics of infliximab in ankylosing spondylitis [18] and inflammatory bowel disease [19], but the determining factor in the concentration-effect relation of infliximab is infliximab itself.

Only one patient was positive for ATI, and this can be explained by the duration of infliximab treatment, 56 months [3 to 76]. Indeed, patients positive for ATI probably had already switched to another treatment because of clinical signs of immunization or secondary failure of treatment. ATI false-negative results may have occurred for sera with detectable infliximab concentrations.

Our study has several limitations. Because it is an open-label observational study, patients may have been influenced by the therapeutic decision, especially those who were notified of an increase in infliximab dosage. Therefore, our results need to be confirmed by a randomized controlled study comparing therapeutic drug monitoring of infliximab with usual care. Also, our approach, based on a single clinical assessment, can be applied only for patients considered to have stable disease activity and not those with acute flare, whose condition requires a rapid therapeutic intervention. Because patients with acute flare were not considered in our study, we cannot extend our strategy to such patients. Because RA activity varies with time, we used a weekly diary to estimate global disease activity between two consecutive infusions to limit this factor. Another limitation of our study is that our population of RA patients had been treated for a long time with infliximab. Whether a dosage adjustment during the early initiation of treatment could be of benefit in terms of response is a challenging question. Since the concentration-effect relationship of infliximab is poorly known, therapeutic drug monitoring will be more difficult to test at treatment initiation. For that reason, our results apply only to RA patients with a relatively long period of treatment, not to patients at their initiation.

## Conclusions

The measurement of serum trough infliximab concentration modifies the therapeutic decision for RA patients and leads to improved control of disease activity. Thus, therapeutic drug monitoring of infliximab may improve the control of disease activity in RA.

## Abbreviations

ATI: antibody toward infliximab; DAS28: disease activity score for 28 joints; ELISA: enzyme-linked immunosorbent assay; RA: rheumatoid arthritis; TNF-α: tumor necrosis factor-alpha; V1, V2, and so on: visit 1, visit 2, and so on.

## Competing interests

DM and J-PV took part in clinical trials as co-investigators for Abbott (Abbott Park, IL, USA), Schering-Plough Corporation (Kenilworth, NJ, USA), Wyeth (Madison, NJ, USA), Roche (Basel, Switzerland), and Bristol-Myers Squibb Company (Princeton, NJ, USA). PG participated in clinical trials as a co-investigator and as a study contributor for Abbott, Schering-Plough Corporation, Wyeth, Roche, and Bristol-Myers Squibb Company and took part in conferences and provided expert reports and advisory services for L.F.B. (Courtabœuf, France), Roche, Schering-Plough Corporation, and Wyeth. GP took part in conferences and provided expert reports and advisory services for L.F.B., Roche, Schering-Plough Corporation, and Wyeth. CM-B had occasional involvement with expert reports for Abbott. All other authors declare that they have no competing interests.

## Authors' contributions

DM and J-CM supervised the study design, performed the statistical analysis, and drafted the manuscript. GP participated in the study design, carried out the immuno assays (infliximab serum concentration), and helped to draft the manuscript. PG participated in the study design and helped to draft the manuscript. CM-B carried out the immuno assays (antibodies toward infliximab serum concentration). ED and J-PV helped to draft the manuscript. All authors read and approved the final manuscript.

## Supplementary Material

Additional file 1 Differences between preliminary and final therapeutic decision. At visit 1 (V1), 24 rheumatoid arthritis (RA) patients received a preliminary therapeutic decision that corresponded to the following options: decrease infliximab dosage; same infliximab dosage plus another intervention; increase infliximab dosage; discontinue infliximab; and switch to another treatment. The final decision (same options) applied at V2 took into account disease activity control and trough infliximab concentration measured at V1 (Table 1). Click here for file
