# Supplementary figures and images for: Infliximab concentration monitoring improves the control of disease activity in rheumatoid arthritis
Source: Arthritis Res Ther. 2009 Nov 25;11(6):R178. doi: 10.1186/ar2867 (PMC3003525; doi:10.1186/ar2867)

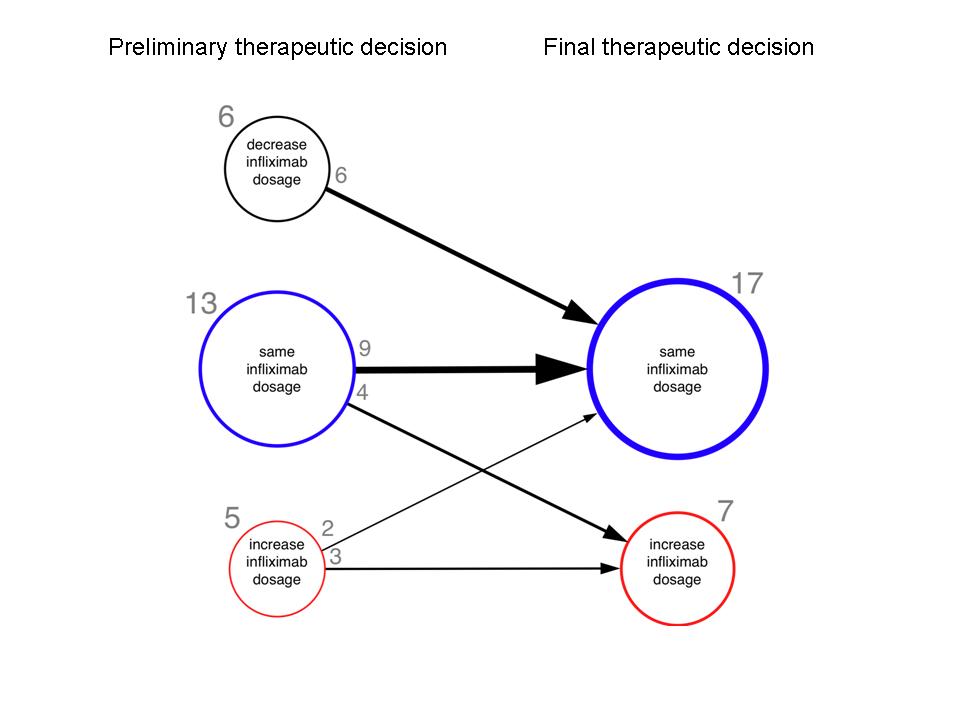

Supplement: Additional file 1 — Differences between preliminary and final therapeutic decision. At visit 1 (V1), 24 rheumatoid arthritis (RA) patients received a preliminary therapeutic decision that corresponded to the following options: decrease infliximab dosage; same infliximab dosage plus another intervention; increase infliximab dosage; discontinue infliximab; and switch to another treatment. The final decision (same options) applied at V2 took into account disease activity control and trough infliximab concentration measured at V1 (Table 1). [file ar2867-S1.JPEG]
